# Simple Modification of a Commercial Laser Triangulation Sensor for Distance Measurement of Slot and Bore Side Surfaces

**DOI:** 10.3390/s21206911

**Published:** 2021-10-18

**Authors:** Jan Hošek, Petr Linduška

**Affiliations:** 1 Faculty of Mechanical Engineering, Czech Technical University in Prague, Technická 4, 166 07 Praha, Czech Republic; plinduska@cmi.cz; 2 Czech Metrology Institute, Okružní 31, 638 00 Brno, Czech Republic

**Keywords:** laser triangulation, hole, slot, distance measurement, beam, diffraction, mirror, edge

## Abstract

The aim of the research is to analyze the possibility of the development and realization of a common laser triangulation sensor arrangement-based probe for the measurement of slots and bore sides with the help of a mirror attachment. The analysis shows the feasibility and limits of the solution with respect to the maximum measurement depth and surface distance measurement working range. We propose two possible solutions: one for maximizing the ratio of the measurement depth to the measured bore size and the second for maximizing the total depth, intended for the measurement of slots and large bore sizes. We analyzed measurement error sources. We found that the errors related to the reflection mirror misalignment can be fully compensated. We proved the validity of the proposed solution with the realization of a commercial laser triangulation sensor-based probe and demonstrated a slot side and a bore side surface distance scanning measurement. The probe working range was assessed with regard to the obscuration effect of optical beams.

## 1. Introduction

Slots and bores are important geometrical features used for a part’s connection or its precise alignment and assembly. Especially in the aerospace, automotive, medical, optical, or generally fine mechanical industry fields, the accuracy and position of such a part’s geometry has to be certain to assure assembly precision and interchangeability. Therefore, a high-accuracy inspection for the slots’ and bores’ parameters is very important.

The metrology and inspection methods for a precise part’s geometry measurement have been extensively developed over the last few decades. Traditionally, contact methods have the advantage of precision and flexibility, but require significant time to reach a data set with a high number of measured points. Particularly a small, complicated, or unknown shape of measured geometry increases the demands for operator skills and leads to increases in the number of environmental factors’ influence on the measurement uncertainty or leads to the development of special kinds of probes [1,2,3]. Due to this, there are new non-contact measurement techniques especially in the case of bore measurement. The standard instrument for precise bore measurement is a dial bore gauge with three balls or segments [4]. A pneumatic micrometer is an alternative for the assessment of machined bores on the shop floor [5]. The advantage of a pneumatic micrometer is high accuracy down to 0.5 μm due to the averaging of the probe to bore surface distance over a large measured surface. On the other hand, it suffers from a small working range usually in dozens of microns. Capacitive sensing probes intended for bore measurement use a similar measured distance averaging principle [6,7,8].

The bore diameter is not the only property that needs to be measured. The inner profiles of engine cylinders and other parts must be measured [9]. Optical instruments such as borescopes and endoscopes are intended for bore inspection, although they may also provide other information [10,11]. However, these are not suitable for measuring the inner profile since the main purpose of them is observation [12]. Some other optical inner diameter and/or profile measurement methods have been proposed on the basis of a circular ring [13,14], structured or diffractive light projection [15,16], and the beam triangulation method using mechanical rotation [17]. The laser triangulation methods are widely used for bore profiling. This is due to the fact that the laser triangulation scanning system can reach micron levels, measure simultaneously, and can be cost-effective compared to other measuring principles. However, for an inner surface’s measurement, a bespoke measuring attachment needs to be constructed [18,19,20,21]. For this reason, the objective of this study is aimed at the possibilities of using a common laser triangulation sensor scheme modified to perform bore and slot sides distance measurement.

The general principle of a laser triangulation sensor is introduced in Section 2. The theoretic consideration of the sensor arrangement able to achieve slot/bore side distance measurement and a corresponding working range are presented in Section 3. We proposed two possible sensor arrangements to fulfill the desired task. The influence of the reflecting mirror inclination angle on the system function is discussed in Section 4. The probe designed for experimental verification is described in Section 5. Section 6 proves the system functionality for vertical surface distance measurement demonstrated with a sample block. The results are focused on the limits of the sensor linearity and scanning range caused by the laser spot imaging beam obscuration by the measured block edge. We determined the beam obscuration level at which the sensor’s distance data are not affected. Finally, Section 7 shows the measurement of the inner walls of bores down to a diameter of 6 mm. All the important findings are discussed at the end of the article.

## 2. Principle of Laser Triangulation Sensor

**Figure 1:**
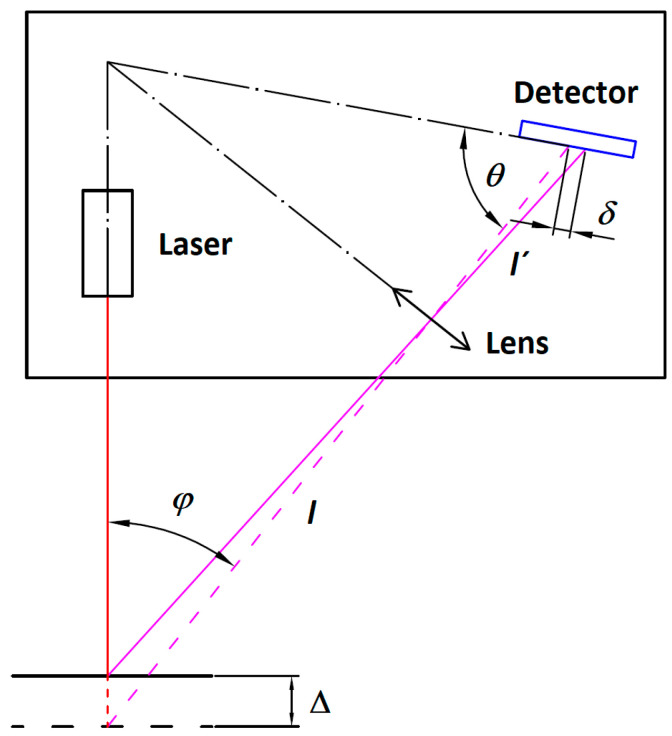
Principle of Laser Triangulation Sensor. Red line represents the focused illumination laser beam. Magenta lines represent laser spot imaging beams through the lens to the sensor’s detector. The detector inclination meets Scheimpflug’s condition.

The general principle of a laser triangulation sensor is shown in Figure 1. 

A focused laser beam projected toward the object’s surface produces a laser spot. As the object’s surface is moved, the laser spot indicates the actual surface position. The light scattered from the laser spot propagates back to the sensor’s imaging lens. An image of the laser spot is formed in the plane of the linear CCD detector. To observe the movement of the object surface along the illumination laser beam, there has to be a non-zero angle *φ* between the laser beam and the sensor’s imaging optics axis. The Scheimpflug’s condition [22]:

*l*tan* φ = l′*tan* θ* (1)

has to be applied in the sensor design thus that the laser spot, moving in a plane inclined to the imaging optics’ axis, will be imaged clearly onto the linear array image detector within the optics’ depth of field. The geometrical relation between the laser spot position, shifted due to the object displacement Δ, and the image displacement *δ,* can be expressed with the triangle similarity principle:

(2) $$\delta =\frac{\Delta l^{\prime }\sin\theta }{l\sin\theta \mp \Delta \sin\left(\varphi +\theta \right)}$$

If the surface moves up from the reference position, “+” in the formula is applied, otherwise, is it “−”. The distance position data are evaluated with the laser spot image center extraction algorithm. Many other factors can interfere with the data acquisition accuracy of the sensor, such as the measurement environment, the surface properties, or other inherent properties of the sensor. Yang et al. analyzed the reason for the laser beam dithering in a laser triangulation sensor and proposed a method for suppressing the dithering impact [23]. Muralikrishnan estimated the influence of different sources of error on a laser spot triangulation probe through a variety of experiments with the focus on slots and channels measurement with an inclined laser probe [24]. He also reported that measurement of the slot edges or sides was difficult, and the measured data were strongly affected by nonlinearity and secondary reflections, even in the case of a small sensor tilt. For this reason, we focus on solving the problem of the slots and bore sides measurement.

## 3. Theory

**Figure 2:**
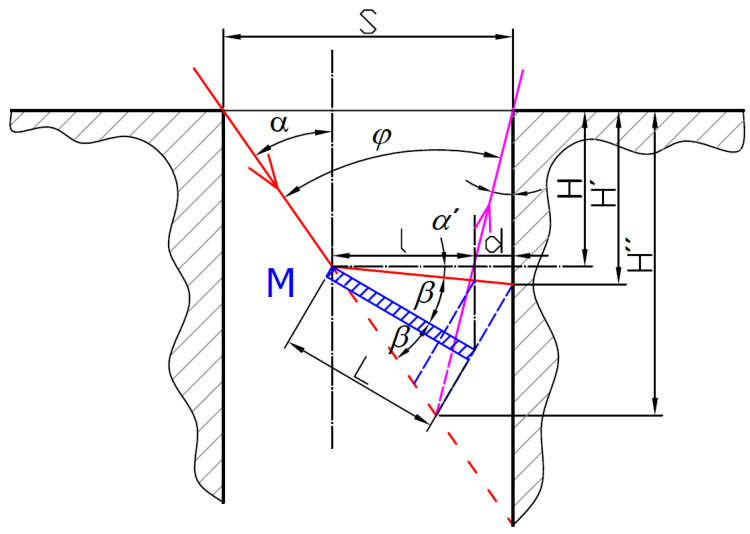
The general situation of laser triangulation probe with mirror *M*. The red arrowed line is the illumination laser beam, magenta arrowed line is the beam imaging the laser spot on the detector.

There is a clear need for laser triangulation measurement of the side surfaces of slots and bores. To ensure the common triangulation sensor’s correct surface distance measurement, the laser illumination beam needs to maintain the normal incidence to the measured surface. This can be achieved with the help of a mirror folding the laser illumination beam and simultaneously reflecting the laser spot back onto the sensor’s detector. The general situation of the laser triangulation probe beams with a mirror *M* enabling the wall distance measurement is shown in Figure 2.

The sensor is characterized by the angle *φ* between the laser illumination beam (IB) and laser spot imaging beam (SIB) in its inherent design. To successfully measure the inner walls of a bore with the size *S*, neither of the beams must be obstructed by the bore. It is clear that this limits the maximum measurement depth *H*′ inside the bore. The maximum depth of the intersection of the illumination and imaging beams *H*″ can be expressed as a triangle altitude:

(3) $$H^{\prime \prime }=\frac{S}{\tan\left(\alpha \right)+\tan\left(\varphi -\alpha \right)}$$

**Figure 3:**
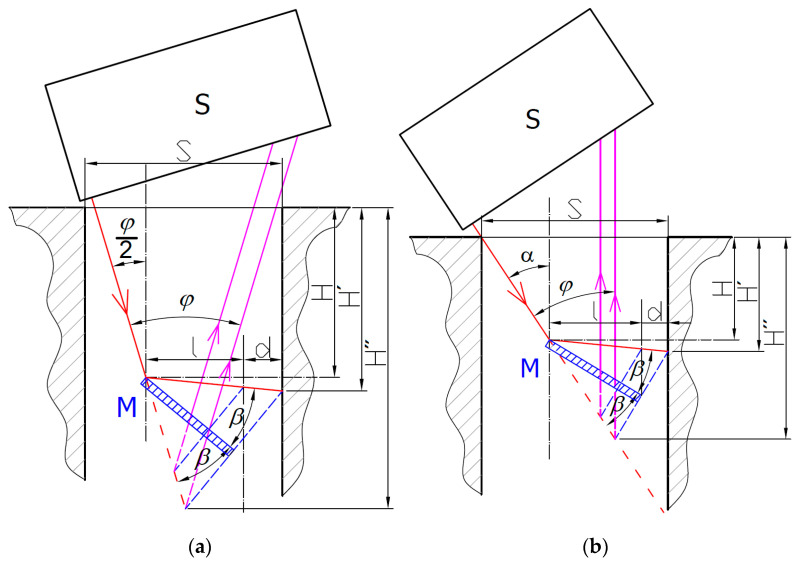
Two possible solutions for the side wall distance measurement with a common laser triangulation sensor S: (**a**) symmetric sensor arrangement (*α* = *φ*/2); (**b**)parallel sensor arrangement (*α* = *φ*).

This relation reaches its maximum when *α* = *φ*/2. This shows that the best sensor inclination is a symmetric illumination and imaging direction with respect to the bore axis. Such a symmetric sensor arrangement (SSA) is shown in Figure 3a. Another solution can be reached for sufficiently wide slots or bores where the size *S* is not a limiting factor. Then, the maximum measurable depth *H*′ is given by the radial size of the mirror *l* from the mirror reflection point and the distance *d* from the mirror tip to the measured surface. The radial size of the mirror simply relates to the mirror’s real functional length *L* through the goniometric function:

(4) $$l=L\cos\left(\beta +\alpha^{\prime }\right)$$

For the SSA arrangement, the depth of the illuminated spot can be expressed as:

(5) $$H^{\prime }=\left(l+d\right)\left(\frac{\tan\left(\alpha \right)+\tan\left(\varphi -\alpha \right)}{\tan\left(\alpha \right)\tan\left(\varphi -\alpha \right)}\left(1-\tan\left(\alpha \right)\left[\cos\left(\alpha^{\prime }\right)\sin\left(2\beta \right)-\sin\left(\alpha^{\prime }\right)\cos\left(2\beta \right)\right]\right)+\tan\left(\alpha^{\prime }\right)-\frac{1}{\tan\left(\alpha \right)}\right)$$

where *β* is the mirror inclination angle with respect to the IB axis. Due to the mirror reflection rule, the reflected beam propagates at the same angle *β*, which may lead to an angle *α*′ between the reflected beam direction and the horizontal axis along which the distance is intended to be measured. The angle *α*′ can be expressed as:

(6) $$\alpha^{\prime }=\frac{\pi }{2}-\alpha -2\beta$$

The derivation of Equation (5) allows us to find the extreme of the maximum depth *H*′, which happens for *α* = *φ* and *α*′ → max. It corresponds to the situation where the SIB is parallel to the bore axis, preventing any light-blocking of the SIB by the bore’s or slot’s upper edge. Such a parallel sensor arrangement (PSA) is shown in Figure 3b.

If we consider the limitation of the IB in the PSA arrangement with the bore or slot size *S*, the maximum reachable measurement depth can be expressed as:

(7) $$H^{\prime }=\frac{S-\left(l+d\right)}{\tan\varphi }+\left(l+d\right)\tan\alpha^{\prime }$$

Despite that fact that the reachable measurement depth *H*′ can be increased with increases of *α*′ angle, this would introduce deviations of the measured laser spot position. For this reason, it is preferred to minimize *α*′ angle to zero to reach a perpendicular incidence to the measured surface. This requires that the optimal mirror angle be equal to:

(8) $$\beta =\frac{90{}^{\circ}-\alpha }{2}$$

where *α* = *φ*/2 for the SSA arrangement and *α* = *φ* for the PSA arrangement.

**Figure 4:**
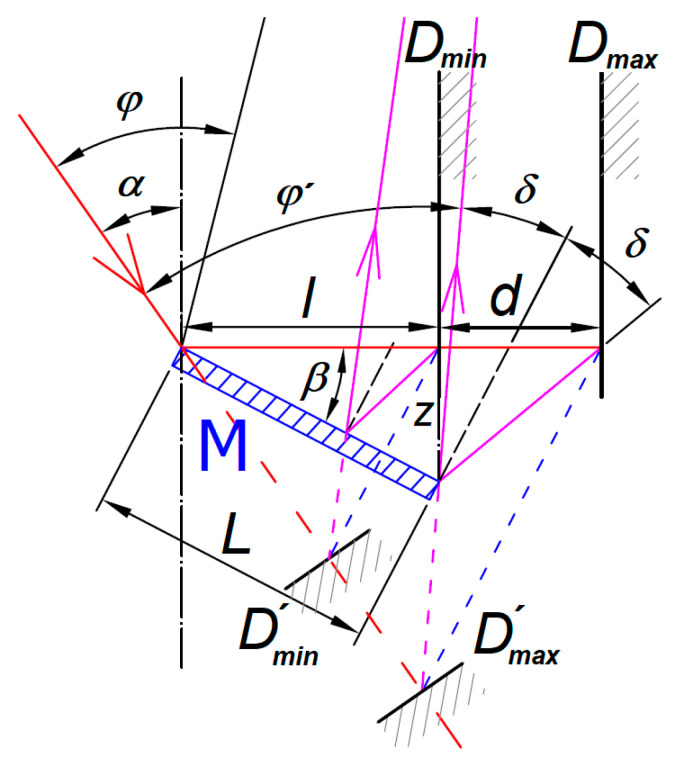
Detail of laser triangulation probe showing minimum *D*_min_ and maximum *D*_max_ positions of the sample surface and its corresponding mirror image positions *D′*_min_ and *D′*_max_. SIB of *D′*_max_ surface image is reflected by the edge of the mirror.

Besides the reachable measurement depth, there is another important parameter of the system: The achievable working range of the sensor *d*. This parameter is not a function of the original sensor working range only, but it is mainly affected by the mirror size *L* and the condition that the laser spot e can be imaged onto the sensor’s detector. As the mirror has a physical dimension, the minimum measured surface distance *D*_min_ cannot be closer than the maximum mirror radial position *l*. According to Figure 4, the maximum working range *d* can be expressed with the help of two triangles. The distance *z* of the mirror is written as:

(9) $$z=L\sin\beta =\frac{d}{\tan\left(\varphi^{\prime }-\alpha +2\delta \right)}$$

where the *δ* angle is:

(10) $$\delta =\beta +\alpha -\varphi^{\prime }$$

and *φ*´ is the angle between the sensor IB and SIB axes at the mirror edge through the sensor’s imaging lens, while the angle *φ* varies along with the sensor’s working range. 

From Equations (9) and (10) we can find the maximum working range *d* between the minimum *D*_min_ and maximum *D*_max_ positions of the measured surface as a function of the mirror length *L* and the mirror angle *β*:

(11) $$d=L\sin\beta \tan\left(2\beta +\alpha -\varphi^{\prime }\right)$$

that can be simplified with the help of Equation (8) to:

(12) $$d=L\frac{\sin\beta }{\tan\varphi^{\prime }}=L\sin\beta \tan\left(\beta +\delta \right)$$

Equation (12) shows that the maximum working range *d* can be linearly increased with increases of the mirror size *L* in front of the reflection point and nonlinearly with increases of the mirror’s and the sensor’s angles. As the mirror angle *β* is related to the sensor angle *φ* through the Relation (8), a longer working range *d* can be reached by using sensors intended for larger working ranges having smaller *φ* angles and using the SSA arrangement. For the same reason, the longest working range *d* can be reached by setting the sensor’s maximum working distance equal to the position *D*_max_ whilst the angle *φ*´ is minimum.

There are reasons why for placing the sensor’s maximum working distance to another position other than the theoretically best position *D*_max_. The Relation (8) do not assume a real size of the sensor’s optics aperture and the laser spot size given by the IB divergence. The real size of the laser spot has to be imaged completely by the mirror close to the edge without the SIB obscuration. Otherwise, the sensor’s laser spot image position algorithm gives an incorrect result. It shortens the maximum working distance compared to the *D*_max_ position. A more realistic usable working range can be estimated by assuming that the 2*δ* angle, shown in Figure 4, corresponds to the angle between the laser spot rim and the corresponding imaging optics aperture edge. In all cases, the mirror position has to be set to assure the sensor’s working range covers all distances between the *D*_min_ and *D*_max_ positions.

## 4. Possible Errors and Optimal Mirror Position

Using a commercially available triangulation sensor is favorable for research due to the possibility of using the sensor distance data without the need for raw data processing, such as the laser spot image centroid search or the sensor nonlinearity correction. This assumption is valid if all the measurement conditions of the sensor are fulfilled, such as, e.g., the measured surface is perpendicular to the direction of the illumination laser beam. For this reason, we searched for properties, which may affect the sensor distance data.

When a single mirror is used for the reflection of both the IB and the SIB it assures that the laser spot is always imaged back into the IB axis under any mirror inclination angle *β*. Another situation arises when the direction of the surface distance measurement is not the same as the reflected illumination laser beam axis. In such a case, there can be found a cosine error that has to be compensated for. A typical task of the surface distance measurement uses the Cartesian coordination system in which the sensor is moved for the surface scanning. If the IB reflected by the mirror is not aligned with one of the coordination system’s axes, the measured distance sensor’s data are affected by that error. There are two main reasons causing the reflected beam’s misalignment:

(A) The reflection mirror inclination angle deviates from the exact value given by Equation (8).
(B) The whole sensor system with the reflection mirror is not aligned with the coordination system used for distance scanning.

Now, we separately analyze the influence of each effect.

### 4.1. Non-Correct Reflection Mirror Inclination Angle

If the mirror angle *β* deviates from the value given by Equation (8) by an angle *ε,* the reflected IB deviates by the double angle 2*ε* and the laser spot is shifted to a new position on the measured surface. The position of the shifted laser spot is mirrored back to the IB axis by the tilted mirror M, which leads to a change of the laser spot position with regards to the original position. The value of the laser spot position change Δ*D* can be expressed with:

(13) $$\cos2\varepsilon =\frac{D}{D+\Delta D}$$

which leads to the laser spot image position error value:

(14) $$\Delta D_{\varepsilon }=D\left(\frac{1}{\cos2\varepsilon }-1\right)$$

It shows that the error caused by an incorrect mirror angle varies not only with the tilt angle *ε* but also depends on the real distance of the measured surface within the sensor working range *d*, because:

(15) D=l+d

### 4.2. Whole Sensor System Rotation

A tilt of the whole system by an angle *γ* with respect to the original IB direction causes the reflected IB to form a shifted laser spot at the measured surface. The position of the shifted laser spot is mirrored back to a new position of the IB axis by the new position of the mirror. Comparing the original laser spot image position and its position with the tilted axis, there is a change of the laser spot image position by the error Δ*D_γ_*. The sensor will detect the surface in a more distant position *D*′, which can be expressed as:

(16) $$D^{\prime }=D+\Delta D_{\gamma }=\frac{D}{\cos\gamma }$$

The corresponding error value Δ*D_γ_* is:

(17) $$\Delta D_{\gamma }=D\left(\frac{1}{\cos\gamma }-1\right)$$

It is obvious that Relations (14) and (17) are similar and differ only with the angle value. It is possible to use it for the error’s compensation under the condition:

(18) $$\Delta D=\Delta D_{\varepsilon }+\Delta D_{\gamma }=0=D\left(\frac{1}{\cos2\varepsilon }-1\right)+D\left(\frac{1}{\cos\gamma }-1\right)$$

The laser spot image position error compensation occurs when:

(19) γ=−2ε

It shows that the mirror inclination error *ε* can be fully compensated by tilting the whole sensor system in the opposite angle direction. This can be used for static applications only. For dynamic scanning applications such as in [25], the compensation tilt of the whole sensor is not feasible, and the sensor distance data have to be corrected numerically with the help of Equation (14).

### 4.3. Measured Surface Inclination

Even if the direction of the reflected IB coincides with the axis of the surface distance measurement, the measured surface can be generally inclined to this axis. An inclination of the measured surface from the perpendicular direction to the IB leads to a shift of the laser spot image maximum intensity point at the laser triangulation sensor detector. The reason is a noncircular laser spot profile caused by an angular section of the Gaussian IB profile from the laser beam waste [26]. Another origin of the surface inclination error is due to non-uniform surface scattering properties. The inclined surface with a Lambertian scattering characteristic shifts the laser spot image centroid as derived in [27]. Fortunately, both these effects can be effectively compensated for even for large surface inclination angles, as in the case of thread profile measurement [28].

## 5. Probe for the Experimental Verification

To prove the functionality and measurement possibilities of vertical surfaces of slots and bores with the standard triangulation sensor scheme, we designed a probe system composed of a common commercial triangulation sensor and an attachment. The attachment of the probe consisted of two parts. The first part was a 3D printed part acting as the sensor holder in the appropriate position with regards to the external coordination system used for the probe scanning motions. The same part was also used for fixing a second part carrying the mirror for the vertical surface distance measurement under SSA arrangement.

As a sensor, we used the Micro-Epsilon 1402-5 laser displacement sensor (1D). The reference distance, i.e., the distance from the sensor body to the start of the measurement range of the laser sensor, was 20 mm, and the measurement range was 5 mm. The linearity of this sensor was ≤0.18% of the full scale, which corresponds to 9 μm. The resolution of the 1402-5 sensor was 1.3 μm, with a 14-bit digital reading without applying averaging. The laser spot diameter varied from 110 μm at the start of the measurement range to 650 μm at the end of the measurement range. The angle *φ* between the IB axis and the SIB axis varies from 38.9° at the start of the measurement range to 33.4° at the end of the measurement range.

**Figure 5:**
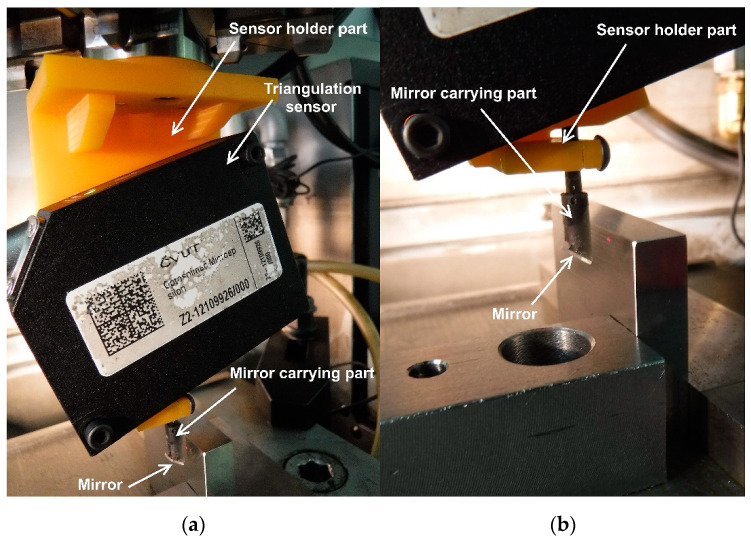
The sensor system probe adapted for vertical surface distance measurement: (**a**) the whole setup view; (**b**) detail of the mirror carrier part.

We used the mirror Edmund Optics 4–6 wave 6.3 mm diameter ground to 4.6 mm width and provided with a straight front edge. The mirror was attached to the carrier part to set the desired mirror inclination angle. The carrier part with the mirror was fixed to the sensor holder part with a clamp joint to allow for the mirror vertical position adjustment. Both the mirror carrier and the sensor holder parts were 3D printed with SLS technique on a PRUSA SL1S 3D printer. We used the Prusa Resign—Tough (Orange and Black) resins (strength limit 52 MPa, Young modulus 1.25 GPa) with a 0.05 mm layer to assure good stiffness, surface quality, and fine precision for setting the mirror position. The whole probe was provided with an EROWA ITS Chucking spigot to allow for its fixing to the machine chuck. We used the 4-axis EDM machine Sodick AP1L to attain the system’s precise scanning motion. The machine provides X, Y, Z translation with uncertainty ± 2 µm and vertical rotation axis indexation. The image of the system under test conditions is shown in Figure 5.

## 6. Vertical Surface Distance Measurement

**Figure 6:**
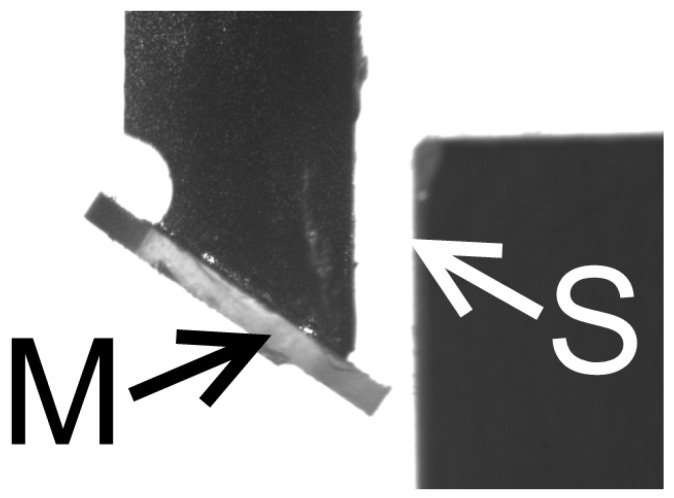
Image of the closest mirror position M to measured surface S in machine coordination position [0, −4], which corresponds to the real mirror edge position [−0.463, −3.915] mm with regards to the top left edge of the sample block.

Our aim was to test the probe for the vertical surface distance measurement demonstrated with a gauge block fixed to the machine’s magnetic table. The first step was to define offsets between the machine coordination system and the real mirror position. We set the mirror edge close to the block side edge and checked its position with 0.4X SilverTL™ Telecentric Lens and DMKUX178 monochrom 6MP Imaging Source camera in the X and *Z*-axis directions with the block edge. Due to safety reasons, the machine coordination system did not coincide with the block edge, but the safety gap was set between the mirror’s edge and the measured surface. This gap may vary when the machine is turned off, thus, we checked the real probe position before the measurement took place. Figure 6 shows the closest mirror’s edge position to the measured block surface in the *X*-axis direction (0.463 mm), which was set before the probe linearity measurement.

The probe mirror was set to an angle *β* = 36.59° with the horizontal axis, and the laser reflection point was adjusted to *L* = 4.51 mm from the mirror’s edge. It allows for reaching a surface distance measurement up to distance *d* = 4.17 mm in front of the mirror’s edge for the sensor’s maximum incident angle *δ* = 20.59° according to Equation (12). In this configuration, the laser incidents the vertical block surface at an altitude of 2.699 mm above the mirror’s edge. Thus, Figure 6 shows the vertical surface distance measurement for the point of the white arrow in the depth of 1.301 mm under the block top surface.

### 6.1. Probe Linearity Measurement

**Figure 7:**
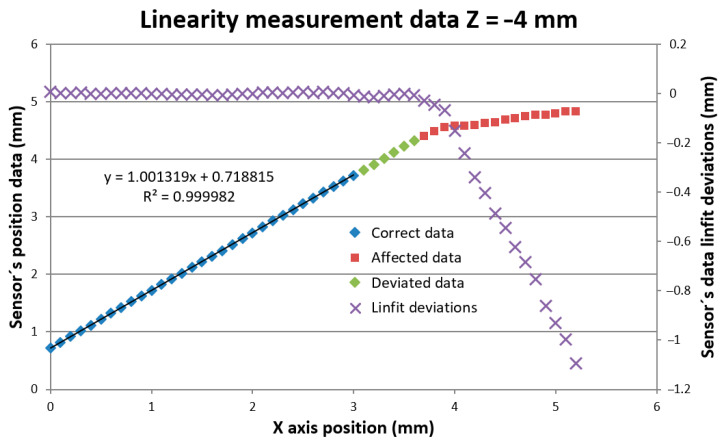
Probe linearity measurement in horizontal *X*-axis motion.

Firstly, the linearity of the sensor distance measurement of the vertical surface of the block was proved. The measurement consisted of distance measurement in the *X*-axis direction perpendicular to the block’s vertical surface. This kind of probe motion assured the laser spot was incident on the fixed part of the block’s surface all the time, and it minimized the influence of the block’s roughness. The surface distance was measured in 0.1 mm steps starting at X = 0 of the machine coordination system position. The sensor distance value was 0.719 mm for X = 0. It corresponds to the minimal offset distance between the mirror edge and the measured surface equal to 0.463 mm. The linearity measurement was performed in four Z positions of the laser spot below the block’s upper edge corresponding to the machine coordination system Z values [−4, −5, −6, −7]. Typical measured data are shown in Figure 7.

The sensor provides position data up to a certain X_err_ distance where it starts indicating data error. Before the error data indication, the sensor provides two kinds of incorrect distance data. We evaluated the limits of the probe’s correct data limit X_corr_ and probe affected data limit X_aff_ positions and summarized that in Table 1.

**Figure 8:**
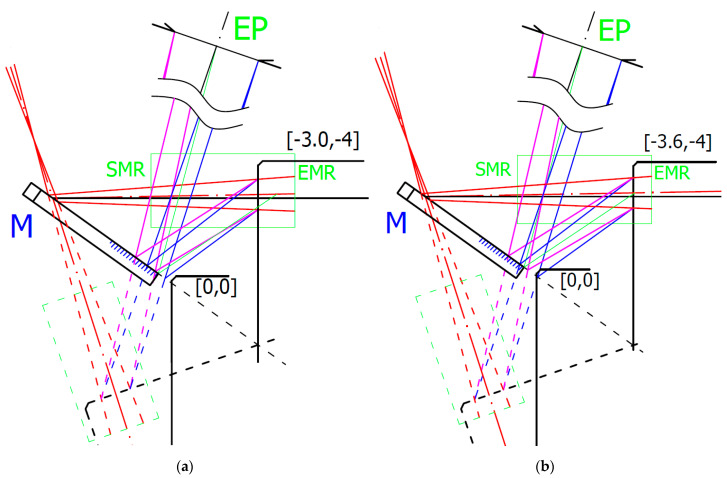
Opto-geometrical model of the probe showing sensor’s optics entrance pupil (EP), mirror M, measured block positions indicated in machine coordinates, and IB in red. Blue and magenta lines correspond to imaging rays of the laser spot sides incident to the opposite rim of the EP. Green ray corresponds to the ray reflecting the center of the laser spot by the edge of the mirror and entering to the center of EP corresponding to the maximum distance d given by Relation (12). The green box indicates start (SMR) and end (EMR) of the measurement range of sensor: (**a**) measured surface in X_corr_ position [−3.0, −4]; (**b**) measured surface in X_aff_ position [−3.6, −4].

To understand the reason for distance data deviation, we made an opto-geometrical model of the probe showing the imaging rays entering the sensor’s optics entrance pupil EP for individual measured positions. Two examples for Z = −4 and the measured surface positions in X_corr_ = −3.0 mm and X_aff_ = −3.6 mm are shown in Figure 8.

Figure 8a shows the last correct surface distance position X_corr_. In the next X position step, the sensor started to deviate from linearity. Within the correct data range, all measurement’s standard deviations were below the 9 μm linearity limit declared by the sensor producer. The correct data range corresponds to the situation where the whole size of the laser spot was imaged onto the sensor’s detector at least by the rim of the sensor’s optics EP. It is shown in Figure 8a, where the EP rim (magenta) ray was at the edge of the reflecting mirror M. Increasing the measured block surface distance leads to loosing of the complete laser spot image showing systematic deviation of the measured data with the average value of more than twice the correct data standard deviation. For an evaluation of the correct distance *d_corr_* measurement limit angles related to rays to the edge of EP *β_e_* and to laser spot edge *δ_e_* have to be considered, and Equation (12) has to be modified to:

(20) $$d_{corr}=(L\cdot \sin\beta_{e}-\frac{D_{S}}{2})\cdot \tan\left(\beta_{e}+\delta_{e}\right)$$

where *D_S_* is the laser spot diameter. It reduced the measured distance to *d_corr_* = 3.456 mm, which was in good agreement with the measured value *d_corr_* = 3 + 0.463 = 3.463 mm.

Figure 8b shows the last affected data of the surface distance position X_aff_. Beyond this X_aff_ position, the sensor data were visibly deviated (see Figure 7) and cannot be considered. This position is shown in Figure 8b. It corresponds to the situation when the mirror reflects just a half of the laser spot that strongly affects the sensor’s processing algorithm. This position corresponds well to the limit of the measured distance *d* = 3.7 + 0.463 = 4.163 mm predicted by the Relation (12). The sensor distance data deviations strongly increased when the mirror stopped reflecting all rays marked in blue.

### 6.2. Vertical Surface Distance Scanning

**Figure 9:**
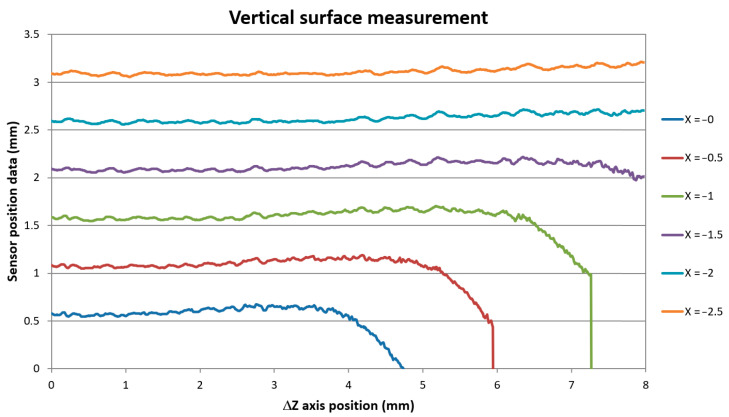
Vertical surface distance scanning data along the *Z*-axis in different *X*-axis positions of the machine coordination system.

Next, we tested the achievable measurement depth of the probe in the SSA configuration. We performed the surface distance measurement with the probe vertically scanning in the *Z*-axis in several different X positions of the mirror in front of the block’s vertical surface. The aim of the measurement was to assess the data repeatability and the depth limit when the data started to be affected by the SIB obscuration by the block’s top edge. We performed measurements in mirror positions from X = 0 mm to X = −2.5 mm, with 0.5 mm steps. The next X positions measurements were not evaluated as they would be affected by beam reflections too close to the mirror edge according to the results of the previous measurement. The measurement of the safety gap showed that X = 0 corresponds to the real front tip of the mirror’s distance of −0.265 mm. We measured the surface with continuous *Z*-axis motion with the velocity of 24 mm/min and sampling frequency of 50 Hz giving data in 0.008 mm steps. We supplemented the continuous measurement with an individual measurement in 0.2 mm steps in the *Z*-axis to achieve the corresponding sensor’s CCD data. Data were gathered from the Z = −4.5 mm position where the sensor provided the distance signal unaffected by the block edge bevel down to Z = −12.5 mm, giving the total of 8 mm *Z*-axis travel. The measured data in the continuous mode are shown in Figure 9.

**Figure 10:**
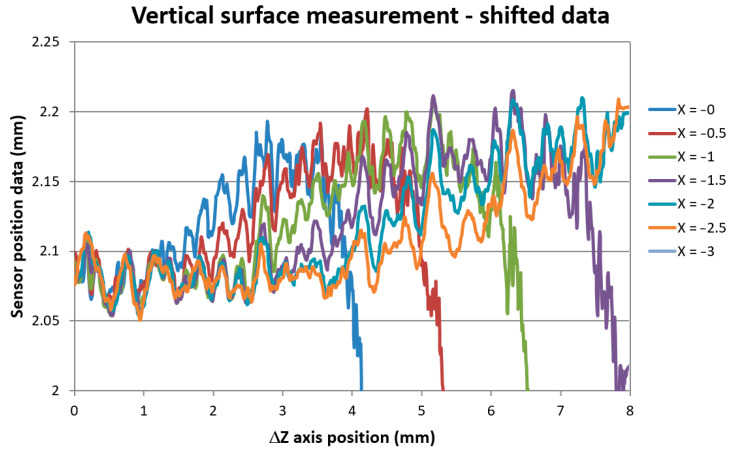
Vertical surface distance scanning data shifted to uniform surface distance indication.

The sensor’s data show the same block surface profile, only shifted to different distance positions. We were able to measure the surface profile down to *Z* = 8 mm axis position, which was our maximum *Z*-axis motion limit given by our probe geometry. This Z position corresponds to the real depth of the laser spot position 9.68 mm below the block’s top surface. The data show the same surface pattern and standard deviation as the distance data scanned with the sensor alone in a standard perpendicular direction of the same surface of the block. The data standard deviation of scans of different sample areas varied from 3 to 9 μm according to the scanned surface roughness and generally increased with the increase of the range of X positions taken for evaluation. The repeatability of individual scans taken along the same scanning line and under the same *X*-axis position was below the 3 micrometer limit as declared by the sensor producer. The data shifted to indicate the same surface distance are shown in Figure 10.

**Figure 11:**
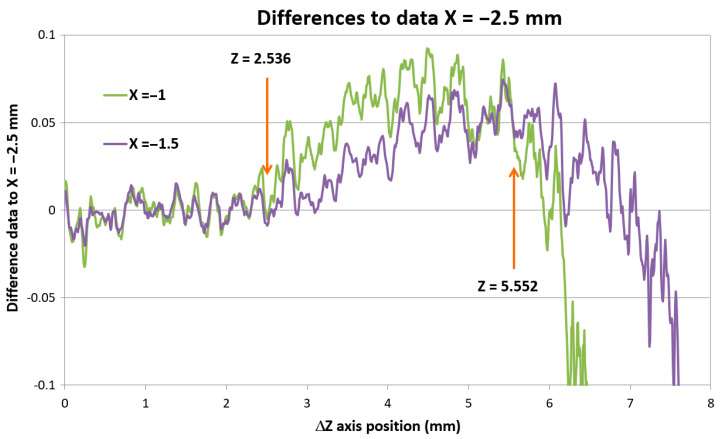
Data differences showing the X = −1 data separation from data taken at distance X = −1.5 mm.

In Figure 11, it can be seen uniform surface distance data measured at all X positions for *Z*-axis position up to approx. ΔZ = 1.1 mm corresponding to the measured real depth of 2.8 mm. The standard deviation of all data was 0.0059 mm within this ΔZ region. Smoothing of scanned surface profiles for different X positions was due to the convolution effect of the rough surface with different laser spot sizes as the IB was divergent.

On closer inspection, it was noticeable that the signals gathered in smaller X distances start to separate from the signal taken in larger X distances at a certain *Z*-axis position. To visualize this effect, Figure 11 shows differences of the data taken at a different X position with regards to data taken in X = −2.5 mm distance.

The reason for this data deviation can be explained with the system opto-geometrical model. We measured the Z positions indicating data deviation from data measured gathered at the next distinct X position. We also measured the Z positions indicating the loss of the laser spot signal. These data are summarized in Table 2.

**Figure 12:**
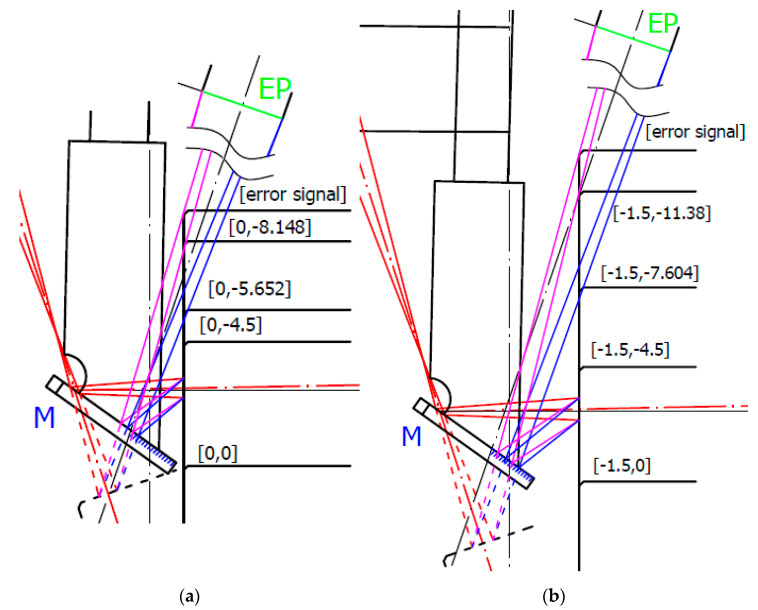
Opto-geometrical model of the probe with measured block’s edge in Z_dev_ and Z_drop_ positions. The scanning origin position was in Z = −4.5. EP is the entrance pupil of the sensor’s optics, M is mirror, and measured block’s positions are indicated in machine coordinates. Blue and magenta lines correspond to imaging rays of the laser spot sides to rims of EP: (**a**) surface positions for X = 0; (**b**) surface positions for X = −1.5.

We built opto-geometrical models of the probe positions corresponding to data deviation Z_dev_ and signal dropping Z_drop_ to visualize the probe imaging beams interaction with the measured block edge. Two examples for X = −0 and X = −1.5 distances are shown in Figure 12.

Figure 12 shows that in both X = −0 and X = −1.5 positions, the data deviation position Z_dev_ corresponds to nearly total obscuration of the rays imaging the laser spot through the rim of the sensor’s EP (indicated in blue). It starts to affect the intensity distribution of the laser spot image, and, consequently, the sensor processing algorithm indicates higher surface distances with a maximum deviation of up to 60 μm. When the block’s edge starts to obscure the SIB rays going through the opposite side of the EP (indicated in magenta), no light from the side part of the laser spot can reach the detector. This causes a strong signal drop to lower distance values. If the block’s edge starts to block nearly all magenta rays, the sensor indicates data processing error.

We evaluated the positions of the measured block’s edge within the SIB to estimate the ratio of the SIB profile blocking leading to the sensor’s data violation. Results are summarized in Table 3.

The evaluation shows that the sensitivity to SIB blocking increases with increases in the measured distance given by the X position, where the beam is blocked by the edge close to the EP. The sensor distance data started to deviate when the SIB was blocked by about 8%, depending on the actual distance to the measured surface. The sensor still provides surface distance data deviated to longer distances up to blocking of about 75% of the SIB.

**Figure 13:**
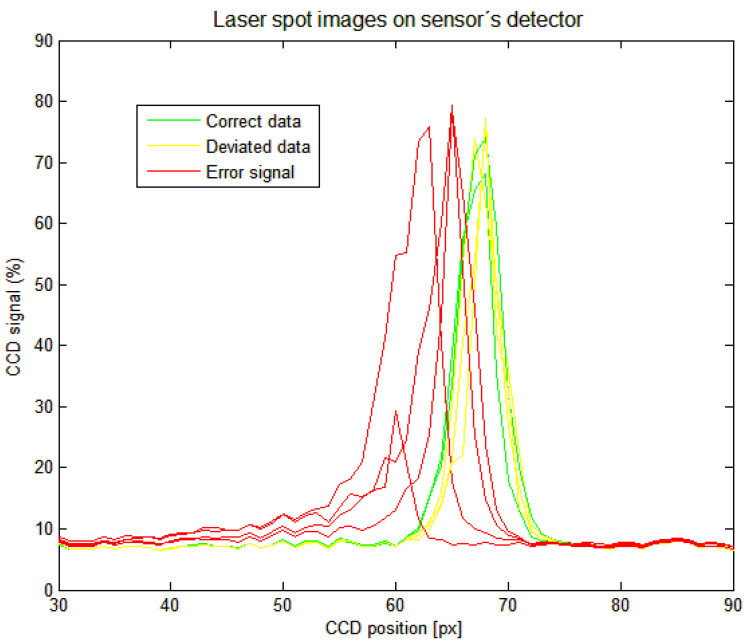
Laser spot profiles gathered by sensor’s linear detector; correct positions data—green, deviated positions data—yellow, error signal positions data—red.

We also analyzed the influence of the SIB on the laser spot image profile at the sensor’s detector in a video mode. The detector signals are shown in Figure 13.

The correct sensor distance data were provided when the laser spot was fully imaged. Deviated distance data result in a narrower laser spot image, and side peaks may appear on the side of the data profile. The sensor’s laser spot profile processing algorithm interprets it as a slight increase in the measured distance. Down and error signals data show one-sided broadening at the bottom of the data profile. The sensor’s laser spot profile processing algorithm interprets this as a decrease in the measured distance. When the peak intensity drops under a defined limit, an error was indicated.

## 7. Bore Side Vertical Surface Distance Measurement

**Figure 14:**
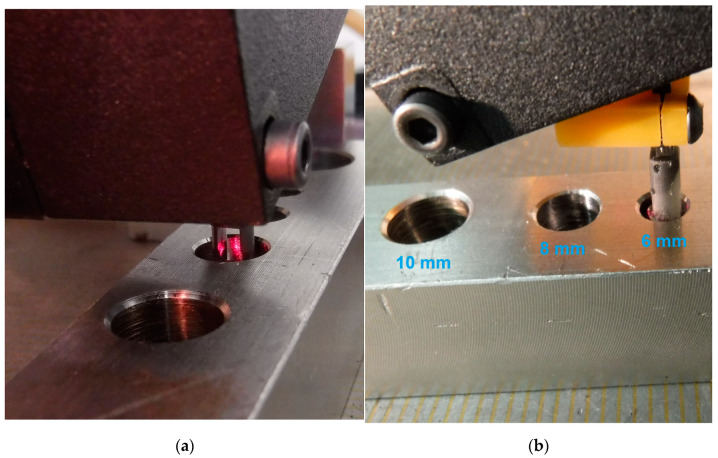
Surface distance measurement with the probe inside the bores: (**a**) measurement inside 8 mm diameter bore; (**b**) measurement inside 6 mm diameter bore.

Finally, we wanted to prove the ability of the probe to measure side profiles inside a hole. The sensor’s attachment with the mirror was designed to have a maximum footprint size of 5.78 mm. It allows us to measure the side profile of a bore down to 6 mm in diameter. We produced a sample with reamed bores of 10 mm, 8 mm, and 6 mm in diameter to test the system’s measurement capability. The measurement of the side surfaces inside the bores is shown in Figure 14.

We gathered data inside the bores along the *Z*-axis under the same conditions as for the block’s side surface measurement. The surface was scanned with a continuous *Z*-axis motion with a velocity of 24 mm/min and a sampling frequency of 50 Hz. The 10 mm and the 8 mm bores were measured in 5 *X*-axis positions separated by 0.5 mm steps. Inside the 6 mm hole, we took just 2 scans separated by *X*-axis distance of 0.2 mm.

**Figure 15:**
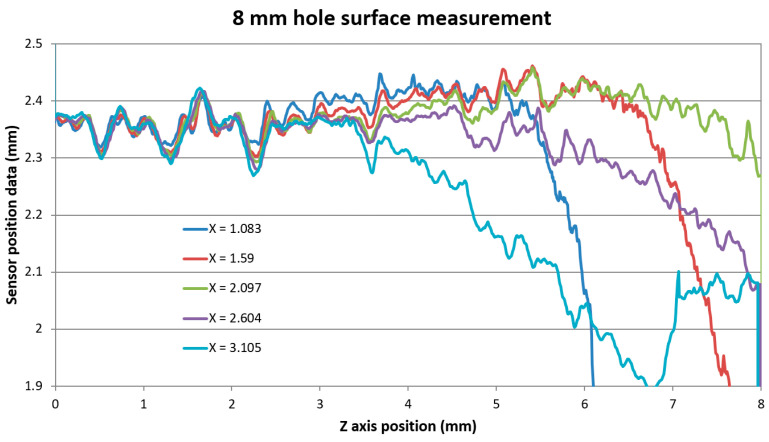
Vertical surface distance scanning data of 8 mm diameter hole shifted to uniform surface distance indication.

The data show similar characteristics as the block’s side surface measurement data. The data uniformed to compensate for the different *X*-axis positions in the case of the 8 mm hole measurement are shown in Figure 15.

**Figure 16:**
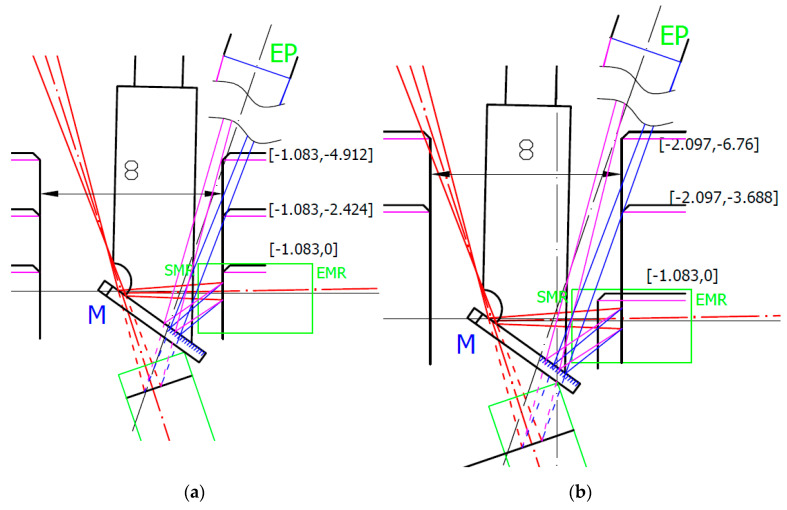
Opto-geometrical model of the probe for 8 mm diameter bore measurement. EP is the sensor’s optics entrance pupil, M is the mirror, measured bore surface positions are indicated in [the sensor distance data, the machine coordinates from the start of the scan]. Blue and magenta lines correspond to imaging rays of the laser spot sides to rims of EP. Green box indicates start (SMR) and end (EMR) of the measurement range of sensor: (**a**) 8 mm bore positions corresponding to start of data scan [−1.083 ,0], start of distance data deviation [−1.083, −2.424], and start of the sensor’s position data drop [−1.083, −4.912]; (**b**) 8 mm bore positions corresponding to start of data scan [−2.097, 0], start of distance data deviation [−2.097, −3.688], and the approximate start of sensor’s position data gradual decreases due to IB obscuration [−2.097, −6.76].

It can be seen that there was a uniform surface distance data measured at all X positions for *Z*-axis positions up to approx. Z = 2.4 mm corresponding to the real measured depth of 3.6 mm inside the bore. The standard deviation of all measured data within the region up to Z = 2.4 mm was evaluated to 0.0079 mm. From this position, individual surface data scanned at a close distance to the probe start to deviate due to the obscuration of the SIB by the bore’s top edge, as shown in Figure 16a. Data scans taken from the distance X = 2.097 mm showed another data drop pattern—the gradual decreases of the evaluated distance. This indicates obscuration of the IB by the bore’s top edge. Figure 16b shows the opto-geometrical model of the probe in position X = 2.097 mm. The probe in this position results in the sensor distance data decreased corresponding to IB obscuration before the sensor distance drops caused by the full SIB obscuration, which would happen at a greater depth.

The bores’ measurement allows us to evaluate the maximum depth of the surface distance measurement inside the bore not affected by the sensor’s distance data deviation. The results are shown in Table 4.

The values of maximum measurable depth were valid for the X position corresponding to the symmetry between the IB and SIB. It led to a weak trend of decreases H_max_/D_hole_ ratio for small bores, while for short measured distances, the *φ*´ angle increased. In the case of a small-bore diameter, the symmetric position might not be possible to achieve due to the limited range of possible probe motion in the *X*-axis direction. In the case of a large-bore diameter, the symmetric position might not be possible to achieve due to the limited maximum surface distance providing unaffected distance measurement. In such a case, the probe in the PSA arrangement could achieve a deeper measurement, despite it is still being limited by the maximum length of the mirror carrier and the overall geometry of the sensor case used.

## 8. Discussion

We proposed two kinds of triangulation sensor-based probe arrangements allowing bores or slots sides distance measurement. The SSA configuration is suitable for the measurement of small-size bores or slots, and the PSA configuration is more suitable for the measurement of large-size bores or slots. Systemic surface distance data deviations were found caused by the loss of the complete laser spot image at the sensor’s detector. It originates in interference of the illumination or imaging beams with mirror or surface edges. It shows the marginal ray model does not predict error-free working range limits correctly in such conditions. Due to that, we modified the relation between the mirror length and the probe working range with respect to the laser spot size, and the results correspond well with the experiment.

We analyzed distance measurements with a probe in the SSA configuration with the help of its opto-geometrical model. Experiments showed that the measured distance data started to deviate when a part of the rays imaging the laser spot through the rim of the sensor’s optics entrance pupil were obscured by the surface’s top edge. There is no evidence of affected distance data up to the obscuration of about 8% of the laser spot imaging beam. A strong distance data deviation occurs when the laser spot imaging beam is blocked by about 75% or more. It may confuse the user as slightly deviated data maintain the surface geometry pattern, but the real surface distance deviates in the scale of tenths of micrometers. 

Finally, we tested the probe for the bores’ side surface measurement. The measurement depth was limited either by the laser spot imaging beam obscuration by the bore’s top edge or by the blocking of the laser illumination beam. Both events are clearly distinguishable due to the different distance data deviation slopes. It allowed us to evaluate the maximum achievable measurement depth in bores, which corresponds to about 65% of the bore diameter. The measurement confirmed a weak trend of ability to reach a greater depth with the increase of the probe distance from the measured surface in the case of larger bore diameters.

## 9. Conclusions

We showed the ability to perform vertical surface distance measurements of slots and bores with a common laser triangulation sensor scheme with a simple mirror attachment in two different arrangements. Analysis of the mirror tilt and the whole probe tilt errors showed that mirror inclination error could be fully compensated for by tilting the whole probe in the opposite angle direction. Otherwise, a numerical correction is needed. 

We evaluated the probe measurement limits given by the mirror length based on a marginal ray model and rim rays’ model. It allowed us to design the appropriate mirror length for desired correct or affected data working range of the probe. It makes no sense to use mirror length, giving a longer probe working range than the working range of the used sensor as the mirror size limits the minimum size of slot or bore to be measured. The minimum mirror size is limited by the size of the illumination laser beam reflected off the mirror and the corresponding minimum reasonable working range. 

Using a commercial triangulation sensor-based probe without additional data processing leads to data deviations when the probe beams are affected by surface or mirror discontinuities as edges or bevels. We showed that the data deviations’ origin could be distinguished with the real beam intensity profiles analysis. It opens the possibility for more complex geometries’ measurement with affected data’s numerical compensation. Our next research will also focus on an extension of the probe’s achievable working range by replacing the mirror with other optical elements.

## Figures and Tables

**Table 1:** Summary of measured surface *X*-axis positions limiting the probe correct data X_corr_, limiting the probe affected data X_aff_, and the error indication X_err_ distances in measured Z-axis positions. St dev row shows the standard deviation of sensor data within the X_corr_ data limit. Avr err row shows average data deviation from correct linfit data within the affected data region from X_corr_ to X_aff_ positions.

Z (mm)	−4	−5	−6	−7
X_corr_ (mm)	3.0	3.0	2.9	2.9
X_aff_ (mm)	3.6	3.5	3.5	3.4
X_err_ (mm)	5.2	5.2	5.1	5.0
St dev (mm)	0.0036	0.0055	0.0043	0.0059
Avr err (mm)	−0.0087	−0.0088	−0.0157	−0.0144

**Table 2:** *Z*-axis position of data deviation Z_dev_ and signal dropping Z_drop_ for data measured at individual X position.

Z-Axis Positions of Data Deviations Relative to the Scanning Origin in Z = −4.5					
(mm)	X = −0	X = −0.5	X = −1	X = −1.5	X = −2
Z_dev_	−1.152	−1.808	−2.536	−3.104	−4.056
Z_drop_	−3.648	−4.616	−5.552	−6.88	-

**Table 3:** Evaluated percentage of the SIB blocking for data deviation Z_dev_ and signal dropping Z_drop_ positions.

The Laser Spot Imaging Beam Blocking (%)				
	X = −0	X = −0.5	X = −1	X = −1.5
Data deviation	8%	9%	10%	8%
Signal drop	75%	74%	74%	75%

**Table 4:** Evaluated maximum depth of the bore side surface distance measurement.

The Maximum Measurable Depth				
D_hole_	6	8	10	(mm)
H_max_	3.9	5.35	6.9	(mm)
H_max_/D_hole_	65	67	69	(%)

## Data Availability

Data are available by contact corresponding author.
